# Supplementary material for: The Small RNA Universe of Capitella teleta
Source: Front Mol Biosci. 2022 Feb 25;9:802814. doi: 10.3389/fmolb.2022.802814 (PMC8915122; doi:10.3389/fmolb.2022.802814)
Supplement: Supplementary file 1 [file DataSheet1.ZIP › Supplement/known/CAPTEscaffold_876_29820.pdf]

Provisional ID	:	CAPTEscaffold_876_29820
Score total	:	5.2
Score for star read(s)	:	-1.3
Score for read counts	:	0
Score for mfe	:	1.9
Score for randfold	:	1.6
Score for cons. seed	:	3
Total read count	:	42345
Mature read count	:	42307
Loop read count	:	2
Star read count	:	36

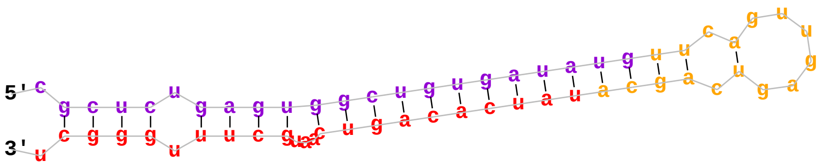

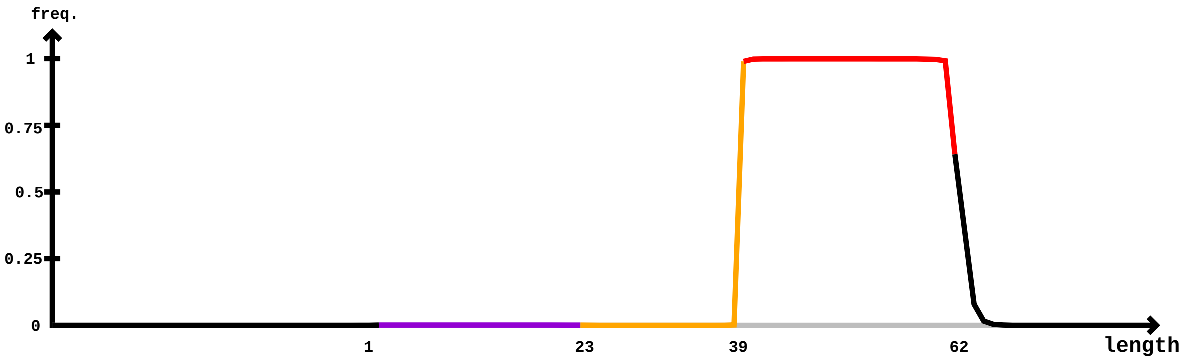

Star Mature

```
5'- uaauugaauugcuugcuuuugcgaauauucugcgcucugaguggcugugauauguucaguugagucagcauaucacagucaaugcuuugggcucagaauauucugcucucuuu   -3'   obs
    uaauugaauugcuugcuuuugcgaauauucugcgcucugaguggcugugauauguucaguugagucagcauaucacagucaaugcuuugggcucagaauauucugcucucuuu         exp
    uaauugaauugcuugcuuuugcgaauauucugcgcucugaguggcugugauauguucaguugagucagcauaucacagucaaugcuuugggcucagaauauucugcucucuuu         known
    ....................((((((((((((.((((.((((((((((((((((((.(......).))))))))))))))...)))).)))).)))))))))).)).......     reads   mm   sample

    ..............................ugcgcucugaguggcugugau..............................................................     3       0    seq
    ..............................ugcgcucugaguggcugugaua.............................................................     3       0    seq
    ................................cgcucugaguggcugugau..............................................................     1       0    seq
    ................................cgcucugaguggcugugaua.............................................................     2       0    seq
    ................................cgcucugaguggcugugauau............................................................     2       0    seq
    ................................cgcucugaguggUugugauaug...........................................................     1       1    seq
    ................................cgcucugaguggcugugauaug...........................................................     29      0    seq
    ................................cgcucugaguggcugugauauguu.........................................................     1       0    seq
    ....................................................uguucaguugagucagcaua.........................................     2       0    seq
    ......................................................uucaguugagucagcauaucacagucaaug.............................     1       0    seq
    ....................................................................cauaucacagucaaugcuuugggc.....................     1       0    seq
    ....................................................................cauaucacagucaaugcuuugggcu....................     3       0    seq
    .....................................................................auaucacagucaaugcuuugggA.....................     1       1    seq
    .....................................................................auaucacagucaaugcuuugggc.....................     12      0    seq
    .....................................................................Cuaucacagucaaugcuuugggc.....................     3       1    seq
    .....................................................................auaucacagucaaugcuuugggcu....................     19      0    seq
    .....................................................................auaucacagucaaugcuuugAgcu....................     1       1    seq
    .....................................................................Guaucacagucaaugcuuugggcu....................     1       1    seq
    .....................................................................Cuaucacagucaaugcuuugggcu....................     3       1    seq
    .....................................................................auaucacagucaaugcuuugggcuc...................     2       0    seq
    .....................................................................auaucacagucaaugcuuugggcuU...................     2       1    seq
    .....................................................................auaucacagucaaugcuuugggcucU..................     5       1    seq
    .....................................................................auaucacagucaaugcuuugggcuca..................     1       0    seq
    .....................................................................auaucacagucaaugcuuugggcucaAa................     1       1    seq
    ......................................................................uaucacagucaaugcuuu.........................     1       0    seq
    ......................................................................uaucacagucaaugcuuug........................     36      0    seq
    ......................................................................uaucacagucaaugcuuuAg.......................     1       1    seq
    ......................................................................uaucacagucaaugcuuugg.......................     41      0    seq
    ......................................................................uaucacagucaaugcuuuAgg......................     1       1    seq
    ......................................................................uaucacagucaaugcuuuggA......................     3       1    seq
    ......................................................................uaucacagucaaugcuuuggg......................     216     0    seq
```

```
                              Star                       Mature
uaauugaauugcuugcuuuugcgaauauucugcgcucugaguggcugugauauguucaguugagucagcauaucacagucaaugcuuugggcucagaauauucugcucucuuu
......................................................................uaucacagucaaugcuuuggC......................	3	1	seq
......................................................................uaucacagucaaAgcuuuggg......................	1	1	seq
......................................................................uaucacagucaaugcuuCgggc.....................	1	1	seq
......................................................................uaucacagucaaugcuuGgggc.....................	1	1	seq
......................................................................uaucacaguGaaugcuuugggc.....................	1	1	seq
......................................................................uauUacagucaaugcuuugggc.....................	6	1	seq
......................................................................uGucacagucaaugcuuugggc.....................	1	1	seq
......................................................................uaucacagucaaugGuuugggc.....................	1	1	seq
......................................................................Naucacagucaaugcuuugggc.....................	2	1	seq
......................................................................uaAcacagucaaugcuuugggc.....................	15	1	seq
......................................................................uaucacaCucaaugcuuugggc.....................	2	1	seq
......................................................................uaucacagucaaugcuuuAggc.....................	48	1	seq
......................................................................uaucacagucaaugcuCugggc.....................	4	1	seq
......................................................................uaucUcagucaaugcuuugggc.....................	1	1	seq
......................................................................uaucacagucaaugcuuuggUc.....................	5	1	seq
......................................................................uaucacagucaaugcuuugggc.....................	14501	0	seq
......................................................................uaucacagucaaugcuuugggU.....................	8	1	seq
......................................................................uaucacagucaaugAuuugggc.....................	6	1	seq
......................................................................uaucacagucaaugcuuuggCc.....................	2	1	seq
......................................................................Aaucacagucaaugcuuugggc.....................	41	1	seq
......................................................................uaucacagucaaugcuuugAgc.....................	15	1	seq
......................................................................Caucacagucaaugcuuugggc.....................	4	1	seq
......................................................................uaucacagucaaugcuuuCggc.....................	1	1	seq
......................................................................uaucGcagucaaugcuuugggc.....................	6	1	seq
......................................................................uauAacagucaaugcuuugggc.....................	3	1	seq
......................................................................uaucacagucaaugcuuugUgc.....................	1	1	seq
......................................................................uaucaAagucaaugcuuugggc.....................	8	1	seq
......................................................................uaucacagucGaugcuuugggc.....................	4	1	seq
......................................................................uaucacagucaaugcCuugggc.....................	7	1	seq
......................................................................uaucacagucaaCgcuuugggc.....................	2	1	seq
......................................................................uaucacagucaauCcuuugggc.....................	3	1	seq
......................................................................uaucacUgucaaugcuuugggc.....................	2	1	seq
......................................................................uaucacagucaaugcuuAgggc.....................	5	1	seq
......................................................................uaucaUagucaaugcuuugggc.....................	1	1	seq
......................................................................uaucacagucaaAgcuuugggc.....................	5	1	seq
......................................................................uaCcacagucaaugcuuugggc.....................	1	1	seq
......................................................................uaucacagucaGugcuuugggc.....................	3	1	seq
......................................................................uaucacagucaaugcuAugggc.....................	3	1	seq
......................................................................uUucacagucaaugcuuugggc.....................	2	1	seq
......................................................................uaucacGgucaaugcuuugggc.....................	3	1	seq
......................................................................uaucacaguAaaugcuuugggc.....................	1	1	seq
......................................................................uaucacagCcaaugcuuugggc.....................	1	1	seq
......................................................................uaucacagucaaugUuuugggc.....................	2	1	seq
......................................................................uaucacagucaaugcAuugggc.....................	4	1	seq
......................................................................uaucacagucaaugcGuugggc.....................	1	1	seq
......................................................................uaucacaguUaaugcuuugggc.....................	8	1	seq
......................................................................uaucacagucaaGgcuuugggc.....................	1	1	seq
......................................................................uaucacagucaaugcuGugggc.....................	1	1	seq
......................................................................uaucacagAcaaugcuuugggc.....................	2	1	seq
......................................................................uaucacagucaauAcuuugggc.....................	2	1	seq
......................................................................uaucacagucaaugcuuugggA.....................	13	1	seq
......................................................................Gaucacagucaaugcuuugggc.....................	9	1	seq
......................................................................uaucacagucaaugcuuuggAc.....................	1	1	seq
......................................................................uaucacagucaauUcuuugggcu....................	1	1	seq
......................................................................uaucacagucaaCgcuuugggcu....................	2	1	seq
......................................................................uaucaAagucaaugcuuugggcu....................	5	1	seq
......................................................................uaucUcagucaaugcuuugggcu....................	5	1	seq
......................................................................uaucacagAcaaugcuuugggcu....................	6	1	seq
......................................................................uaucacaAucaaugcuuugggcu....................	5	1	seq
......................................................................uaucacagGcaaugcuuugggcu....................	1	1	seq
......................................................................uGucacagucaaugcuuugggcu....................	2	1	seq
......................................................................uaucacaCucaaugcuuugggcu....................	3	1	seq
......................................................................uaucacagucaGugcuuugggcu....................	5	1	seq
......................................................................uaucacagucaaugcuuuUggcu....................	2	1	seq
......................................................................uaucacagucaauCcuuugggcu....................	1	1	seq
......................................................................uaucacagucaaugcuuuCggcu....................	1	1	seq
......................................................................uaucacagucaaugcuuugggcG....................	72	1	seq
......................................................................uaucacaguNaaugcuuugggcu....................	1	1	seq
......................................................................uaucaGagucaaugcuuugggcu....................	2	1	seq
......................................................................uauGacagucaaugcuuugggcu....................	4	1	seq
```

```
                                Star                                Mature
uaauugaauugcuugcuuuugcgaauauucugcgcucugaguggcugugauauguucaguugagucagcauaucacagucaaugcuuugggcucagaauauucugcucucuuu
......................................................................uaucacagucaaugcAuugggcu....................  13     1  seq
......................................................................uaucacagucaaugcuuugggAu....................  5      1  seq
......................................................................Naucacagucaaugcuuugggcu....................  8      1  seq
......................................................................uaucacagucaaugcCuugggcu....................  10     1  seq
......................................................................uaucacagucaaugcuuuggUcu....................  1      1  seq
......................................................................uaucaUagucaaugcuuugggcu....................  3      1  seq
......................................................................Caucacagucaaugcuuugggcu....................  5      1  seq
......................................................................Gaucacagucaaugcuuugggcu....................  9      1  seq
......................................................................uaucacagucaaugcuCugggcu....................  4      1  seq
......................................................................uaucacagucaaugcuuCgggcu....................  3      1  seq
......................................................................uaucacagucaaugUuuugggcu....................  1      1  seq
......................................................................uaucacaguGaaugcuuugggcu....................  3      1  seq
......................................................................uaucacagucaaugcuAugggcu....................  4      1  seq
......................................................................uaucacagucaaugcuuugCgcu....................  1      1  seq
......................................................................uaucacagucaaugcuuugggcA....................  64     1  seq
......................................................................uaucacGgucaaugcuuugggcu....................  6      1  seq
......................................................................uauAacagucaaugcuuugggcu....................  1      1  seq
......................................................................uaucacagucaaugcuuugggcu....................  19686  0  seq
......................................................................uaucacagucaaugcuuuAggcu....................  83     1  seq
......................................................................uaucacagucGaugcuuugggcu....................  4      1  seq
......................................................................uaucacagucaaugcuGugggcu....................  1      1  seq
......................................................................uaucacagucaaugcuuGgggcu....................  3      1  seq
......................................................................uaucacaguAaaugcuuugggcu....................  9      1  seq
......................................................................uaucacagucaaugcuuuggAcu....................  3      1  seq
......................................................................uaucacagucaauAcuuugggcu....................  3      1  seq
......................................................................uaucacCgucaaugcuuugggcu....................  2      1  seq
......................................................................uaCcacagucaaugcuuugggcu....................  3      1  seq
......................................................................uaucacagucaaugcuuugUgcu....................  3      1  seq
......................................................................uaucacagucaaugcuuugggcC....................  4      1  seq
......................................................................uaucGcagucaaugcuuugggcu....................  4      1  seq
......................................................................Aaucacagucaaugcuuugggcu....................  77     1  seq
......................................................................uauUacagucaaugcuuugggcu....................  12     1  seq
......................................................................uaucacagucUaugcuuugggcu....................  1      1  seq
......................................................................uaucacagucaaugcuuugAgcu....................  15     1  seq
......................................................................uaucacagucaaugcGuugggcu....................  1      1  seq
......................................................................uaAcacagucaaugcuuugggcu....................  20     1  seq
......................................................................uaucacaUucaaugcuuugggcu....................  2      1  seq
......................................................................uaucacagucaUugcuuugggcu....................  2      1  seq
......................................................................uaucacagucaaugcuuAgggcu....................  8      1  seq
......................................................................uaucacUgucaaugcuuugggcu....................  6      1  seq
......................................................................uaucacaguUaaugcuuugggcu....................  7      1  seq
......................................................................uaucacagucaaugAuuugggcu....................  6      1  seq
......................................................................uaucacagucaaAgcuuugggcu....................  8      1  seq
......................................................................uaucacagucaaGgcuuugggcu....................  1      1  seq
......................................................................uaucacagCcaaugcuuugggcu....................  3      1  seq
......................................................................uNucacagucaaugcuuugggcu....................  1      1  seq
......................................................................uaucacagucaaugGuuugggcu....................  1      1  seq
......................................................................uaucacagucaaugcuuugggUuc...................  1      1  seq
......................................................................uaAcacagucaaugcuuugggcuc...................  1      1  seq
......................................................................uaucacagucaaugcuuugAgcuc...................  3      1  seq
......................................................................uaucacagucaaugcuuugggcuc...................  2701   0  seq
......................................................................uaucacUgucaaugcuuugggcuc...................  1      1  seq
......................................................................uaucacagAcaaugcuuugggcuc...................  1      1  seq
......................................................................uaucacagucaaugcuuuAggcuc...................  10     1  seq
......................................................................uaucacagucaaugcuuAgggcuc...................  2      1  seq
......................................................................uaucacaguAaaugcuuugggcuc...................  1      1  seq
......................................................................uaucacagucaaugcuuugggcuA...................  11     1  seq
......................................................................uaucacagucaaugUuuugggcuc...................  1      1  seq
......................................................................uaucacagucaaugcuuugggcuG...................  1      1  seq
......................................................................uaucacaguUaaugcuuugggcuc...................  2      1  seq
......................................................................Naucacagucaaugcuuugggcuc...................  1      1  seq
......................................................................uaucacagucaauUcuuugggcuc...................  2      1  seq
......................................................................uauUacagucaaugcuuugggcuc...................  2      1  seq
......................................................................uaucGcagucaaugcuuugggcuc...................  1      1  seq
......................................................................uaucacagucaaAgcuuugggcuc...................  4      1  seq
......................................................................uaucacagucaaugcuuCgggcuc...................  2      1  seq
......................................................................uaucacagucaaugcGuugggcuc...................  1      1  seq
......................................................................Caucacagucaaugcuuugggcuc...................  1      1  seq
......................................................................uaucUcagucaaugcuuugggcuc...................  1      1  seq
......................................................................uaucacagucaaugcuuugggcAc...................  1      1  seq
```

Star Mature

```
uaauugaauugcuugcuuuugcgaauauucugcgcucugaguggcugugauauguucaguugagucagcauaucacagucaaugcuuugggcucagaauauucugcucucuuu
......................................................................uaucacagucaaugcAuugggcuc...................    1     1    seq
......................................................................Aaucacagucaaugcuuugggcuc...................    13    1    seq
......................................................................uaucacGgucaaugcuuugggcuc...................    3     1    seq
......................................................................uaucacagucaaugcuAugggcuc...................    2     1    seq
......................................................................uaucacagucaaugAuuugggcuc...................    3     1    seq
......................................................................uaucacagucaaugcuuugggAuc...................    1     1    seq
......................................................................uaucacagucaaugcuuugggcuU...................    477   1    seq
......................................................................uaucacagucaaugAuuugggcuca..................    1     1    seq
......................................................................Aaucacagucaaugcuuugggcuca..................    3     1    seq
......................................................................uaucaUagucaaugcuuugggcuca..................    1     1    seq
......................................................................uaucacagucaaugcuuugggcuca..................    1344  0    seq
......................................................................uaucacagucaaugcuuugggcucU..................    1232  1    seq
......................................................................uaucacagucaaugcuuugggcucG..................    7     1    seq
......................................................................uauUacagucaaugcuuugggcuca..................    1     1    seq
......................................................................uaucacagucaaugcuuuggCcuca..................    1     1    seq
......................................................................uaucGcagucaaugcuuugggcuca..................    1     1    seq
......................................................................uauAacagucaaugcuuugggcuca..................    1     1    seq
......................................................................uaucacagucaaugcuuuggUcuca..................    1     1    seq
......................................................................uaucacagucaaugcuuCgggcuca..................    1     1    seq
......................................................................uaucacagucaaugcuuugAgcuca..................    1     1    seq
......................................................................uaucacagucaauAcuuugggcuca..................    1     1    seq
......................................................................uaucacagucaaugcAuugggcuca..................    2     1    seq
......................................................................uaucacagucaaugcuuugggcuUa..................    5     1    seq
......................................................................uaucacagucaaugcCuugggcuca..................    1     1    seq
......................................................................uUucacagucaaugcuuugggcuca..................    1     1    seq
......................................................................uaucacagAcaaugcuuugggcuca..................    1     1    seq
......................................................................uaAcacagucaaugcuuugggcuca..................    2     1    seq
......................................................................uaucacagucaaugcuuuAggcuca..................    4     1    seq
......................................................................uaucacagucaaugcuuugggcucC..................    14    1    seq
......................................................................Naucacagucaaugcuuugggcuca..................    1     1    seq
......................................................................uaucacagucaaugcuuugggcucaC.................    11    1    seq
......................................................................uaucacagucaaugcuuugggcucaA.................    47    1    seq
......................................................................uaucacagucaaugcuuugggcucag.................    2     0    seq
......................................................................uaucacagucaaugcuuugggcucaU.................    460   1    seq
......................................................................uaucacagucaaugcuuugggcucaUa................    4     1    seq
......................................................................uaucacagucaaugcuuugggcucagU................    1     1    seq
......................................................................uaucacagucaaugcuuugggcucaAa................    91    1    seq
......................................................................uaucacagucaaugcuuugggcucaCa................    1     1    seq
......................................................................uaucacagucaaugcuuugggcucaAaa...............    50    1    seq
......................................................................uaucacagucaaugcuuugggcucaAaau..............    2     1    seq
.......................................................................aucacagucaaugcuuugggc.....................    50    0    seq
.......................................................................Uucacagucaaugcuuugggc.....................    1     1    seq
.......................................................................aucacagucaaugcuuugggcA....................    2     1    seq
.......................................................................aucacaCucaaugcuuugggcu....................    1     1    seq
.......................................................................Gucacagucaaugcuuugggcu....................    2     1    seq
.......................................................................aucacagucaaugcuuugggcu....................    213   0    seq
.......................................................................aucacagucaaugcuuugggcuc...................    45    0    seq
.......................................................................aucacagucaaugcuuugggcuU...................    8     1    seq
.......................................................................aucacagucaaugcuuugggcucU..................    13    1    seq
.......................................................................aucacagucaaugcuuugggcuca..................    19    0    seq
.......................................................................aucacagucaaugcuuugggcucaA.................    1     1    seq
.......................................................................aucacagucaaugcuuugggcucaU.................    3     1    seq
........................................................................ucacagucaaugcuuugggc.....................    1     0    seq
........................................................................ucacagucaaugcuuugggcu....................    14    0    seq
........................................................................ucacagucaaugcuuugggcuc...................    5     0    seq
........................................................................ucacagucaaugcuuugggcuca..................    3     0    seq
........................................................................ucacagucaaugcuuugggcucU..................    1     1    seq
........................................................................ucacagucaaugcuuugggcucag.................    3     0    seq
........................................................................ucacagucaaugcuuugggcucagU................    1     1    seq
........................................................................ucacagucaaugcuuugggcucaga................    4     0    seq
..........................................................................acagucaaugcuuugggcu....................    1     0    seq
..........................................................................acagucaaugcuuugggcuc...................    1     0    seq
...........................................................................cagucaaugcuuugggcuca..................    2     0    seq
```
